# 2-[(*E*)-(2,4-Dimethyl­phen­yl)imino­meth­yl]phenol

**DOI:** 10.1107/S1600536811026110

**Published:** 2011-07-06

**Authors:** Hoong-Kun Fun, Ching Kheng Quah, S. Viveka, D. J. Madhukumar, G. K. Nagaraja

**Affiliations:** a X-ray Crystallography Unit, School of Physics, Universiti Sains Malaysia, 11800 USM, Penang, Malaysia; b Department of Chemistry, Mangalore University, Karnataka, India

## Abstract

The asymmetric unit of the title compound, C_15_H_15_NO, contains two independent mol­ecules, both of which exist in *trans* configurations with respect to the C=N bonds [1.278 (2) and 1.279 (2) Å]. In each mol­ecule, intra­molecular O—H⋯N hydrogen bonds generate *S*(6) ring motifs. In one mol­ecule, the benzene rings form a dihedral angle of 13.38 (9)°, while in the other mol­ecule the dihedral angle is 30.60 (10)°. In the crystal, the two independent mol­ecules are linked *via* weak inter­molecular C—H⋯O hydrogen bonds.

## Related literature

For general background to and the pharmacological activity of Schiff base compounds, see: Gallant *et al.* (2004 ▶); Kulkarni (1975 ▶); Zhao *et al.* (1988 ▶); Ma & Zhao (1988 ▶). For a related structure, see: Fun *et al.* (2011 ▶). For hydrogen-bond motifs, see: Bernstein *et al.* (1995 ▶). For standard bond-length data, see: Allen *et al.* (1987 ▶).
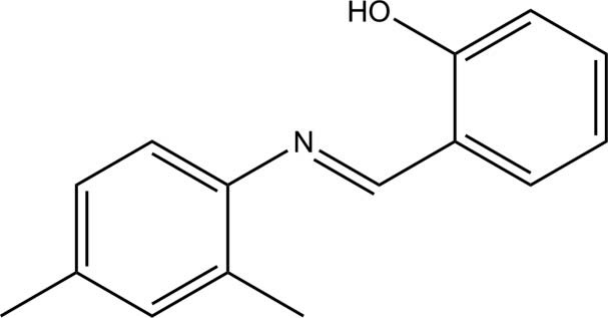


## Experimental

### 

#### Crystal data

C_15_H_15_NO
*M* *_r_* = 225.28
Orthorhombic, 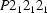

*a* = 7.3161 (4) Å
*b* = 12.0287 (7) Å
*c* = 28.1634 (15) Å
*V* = 2478.5 (2) Å^3^
*Z* = 8
Mo *K*α radiation
μ = 0.08 mm^−1^
*T* = 296 K
0.51 × 0.35 × 0.32 mm

#### Data collection

Bruker SMART APEXII DUO CCD area-detector diffractometer
Absorption correction: multi-scan (*SADABS*; Bruker, 2009 ▶) *T*_min_ = 0.963, *T*_max_ = 0.976
39373 measured reflections
4117 independent reflections
3381 reflections with *I* > 2σ(*I*)
*R*_int_ = 0.027

#### Refinement

*R*[*F* ^2^ > 2σ(*F* ^2^)] = 0.040
*wR*(*F* ^2^) = 0.122
*S* = 1.02
4117 reflections
319 parameters
H atoms treated by a mixture of independent and constrained refinement
Δρ_max_ = 0.19 e Å^−3^
Δρ_min_ = −0.13 e Å^−3^

### 

Data collection: *APEX2* (Bruker, 2009 ▶); cell refinement: *SAINT* (Bruker, 2009 ▶); data reduction: *SAINT*; program(s) used to solve structure: *SHELXTL* (Sheldrick, 2008 ▶); program(s) used to refine structure: *SHELXTL*; molecular graphics: *SHELXTL*; software used to prepare material for publication: *SHELXTL* and *PLATON* (Spek, 2009 ▶).

## Supplementary Material

Crystal structure: contains datablock(s) global, I. DOI: 10.1107/S1600536811026110/lh5275sup1.cif

Structure factors: contains datablock(s) I. DOI: 10.1107/S1600536811026110/lh5275Isup2.hkl

Supplementary material file. DOI: 10.1107/S1600536811026110/lh5275Isup3.cml

Additional supplementary materials:  crystallographic information; 3D view; checkCIF report

## Figures and Tables

**Table 1:** Hydrogen-bond geometry (Å, °)

*D*—H⋯*A*	*D*—H	H⋯*A*	*D*⋯*A*	*D*—H⋯*A*
O1*A*—H1*OA*⋯N1*A*	0.88 (2)	1.80 (2)	2.5854 (19)	147 (2)
O1*B*—H1*OB*⋯N1*B*	0.90 (2)	1.82 (2)	2.604 (2)	145 (2)
C5*A*—H5*AA*⋯O1*B*^i^	0.93	2.56	3.455 (2)	162

Symmetry code: (i) 
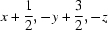
.

**Fig. 1.**
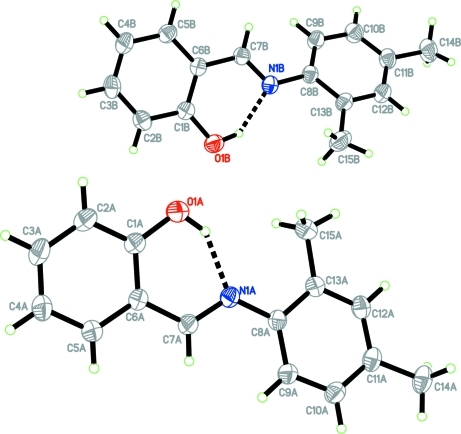
The molecular structure of the title compound showing 30% probability displacement ellipsoids for non-H atoms. Intramolecular hydrogen bonds are shown as dashed lines.

**Fig. 2.**
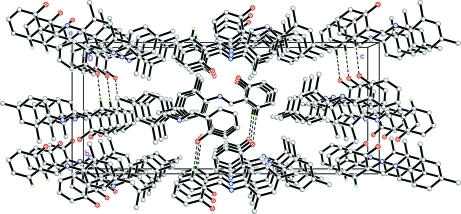
Part of the crystal structure of the title compound, viewed along the a axis. H atoms not involved in hydrogen bonds (dashed lines) have been omitted for clarity.

## supplementary crystallographic information

### Comment

During the last 50 years, a vast number of structural studies on Schiff bases derived from hydroxyaryl aldehydes have been studied. Schiff bases can be synthesized from an aromatic amine and a carbonyl compound in a nucleophilic addition to a hemiaminal followed by elimination of water to the imine (Gallant *et al.*, 2004). These Schiff bases have shown varied redox and electrical behaviors, depending on the involvement of active coordination sites (Kulkarni, 1975; Zhao *et al.*, 1988; Ma & Zhao, 1988). Among the organic reagents actually used, Schiff bases possess excellent characteristics, structural similarities with natural biological substances, relatively simple preparation procedures and the synthetic flexibility that enables the design of suitable structural properties.

The asymmetric unit contains two independent molecules (Fig. 1), *A* and *B*. Both molecules exist in *trans* configurations with respect to the C7═N1 bonds [C7A═N1A = 1.278 (2) Å, C7B═N1B = 1.279 (2) Å]. The molecular structure is stabilized by intramolecular O1A–H1OA···N1A and O1B–H1OB···N1B hydrogen bonds (Table 1) which generate *S*(6) ring motifs (Fig. 1, Bernstein *et al.*, 1995). Bond lengths (Allen *et al.*, 1987) and angles are within normal ranges and are comparable to a related structure (Fun *et al.*, 2011). In molecule *A*, the benzene rings (C1A-C6A and C8A-C13A) form a dihedral angle of 13.38 (9)°. The corresponding dihedral angle for molecule *B* is 30.60 (10)°.

In the crystal structure, Fig. 2, molecules *A* are linked to molecules *B**via* weak intermolecular C5A–H5AA···O1B^i^ hydrogen bonds (Table 1) into pairs.

### Experimental

A mixture of salicylaldehyde (0.01 mol) and 2,4 dimethyl aniline (0.01 mol) in presence of glacial acetic acid (0.5 mL) in ethanol (25 mL) was refluxed gently for 4-5 h. The reaction was monitored by TLC. After completion of the reaction, the reaction mixture was poured into a beaker containing crushed ice. The precipitate thus obtained was filtered, dried and recrystallized from ethanol. Yield: 80%, *m.p.* 425-428 K.

### Refinement

H1OA and H1OB atoms were located in a difference Fourier map and refined freely [O1A–H1OA = 0.88 (3) Å, O1B–H1OB = 0.89 (2) Å]. The remaining H atoms were positioned geometrically and refined using a riding model with C–H = 0.93 or 0.96 Å and *U*_iso_(H) = 1.2 or 1.5 *U*_eq_(C). A rotating-group model was applied for the methyl groups. The highest residual electron density peak is located at 0.72 Å from C7A and the deepest hole is located at 1.28 Å from C12B. In the absence of significant anomalous dispersion, 3161 Friedel pairs were merged for the final refinement.

### Crystal data

**Table:**

C_15_H_15_NO	*F*(000) = 960
*M**_r_* = 225.28	*D*_x_ = 1.207 Mg m^−^^3^
Orthorhombic, *P*2_1_2_1_2_1_	Mo *K*α radiation, λ = 0.71073 Å
Hall symbol: P 2ac 2ab	Cell parameters from 9995 reflections
*a* = 7.3161 (4) Å	θ = 2.8–29.8°
*b* = 12.0287 (7) Å	µ = 0.08 mm^−^^1^
*c* = 28.1634 (15) Å	*T* = 296 K
*V* = 2478.5 (2) Å^3^	Block, yellow
*Z* = 8	0.51 × 0.35 × 0.32 mm

### Data collection

**Table:**

Bruker SMART APEXII DUO CCD area-detector diffractometer	4117 independent reflections
Radiation source: fine-focus sealed tube	3381 reflections with *I* > 2σ(*I*)
graphite	*R*_int_ = 0.027
φ and ω scans	θ_max_ = 30.1°, θ_min_ = 1.8°
Absorption correction: multi-scan (*SADABS*; Bruker, 2009)	*h* = −10→10
*T*_min_ = 0.963, *T*_max_ = 0.976	*k* = −16→16
39373 measured reflections	*l* = −39→39

### Refinement

**Table:**

Refinement on *F*^2^	Primary atom site location: structure-invariant direct methods
Least-squares matrix: full	Secondary atom site location: difference Fourier map
*R*[*F*^2^ > 2σ(*F*^2^)] = 0.040	Hydrogen site location: inferred from neighbouring sites
*wR*(*F*^2^) = 0.122	H atoms treated by a mixture of independent and constrained refinement
*S* = 1.02	*w* = 1/[σ^2^(*F*_o_^2^) + (0.0711*P*)^2^ + 0.1832*P*] where *P* = (*F*_o_^2^ + 2*F*_c_^2^)/3
4117 reflections	(Δ/σ)_max_ = 0.001
319 parameters	Δρ_max_ = 0.19 e Å^−^^3^
0 restraints	Δρ_min_ = −0.13 e Å^−^^3^

### Special details

**Table:**

Geometry. All esds (except the esd in the dihedral angle between two l.s. planes) are estimated using the full covariance matrix. The cell esds are taken into account individually in the estimation of esds in distances, angles and torsion angles; correlations between esds in cell parameters are only used when they are defined by crystal symmetry. An approximate (isotropic) treatment of cell esds is used for estimating esds involving l.s. planes.
Refinement. Refinement of F^2^ against ALL reflections. The weighted R-factor wR and goodness of fit S are based on F^2^, conventional R-factors R are based on F, with F set to zero for negative F^2^. The threshold expression of F^2^ > 2sigma(F^2^) is used only for calculating R-factors(gt) etc. and is not relevant to the choice of reflections for refinement. R-factors based on F^2^ are statistically about twice as large as those based on F, and R- factors based on ALL data will be even larger.

### Fractional atomic coordinates and isotropic or equivalent isotropic displacement parameters (Å^2^)

**Table:**

	*x*	*y*	*z*	*U*_iso_*/*U*_eq_	
O1A	0.9304 (2)	0.77108 (12)	−0.04120 (5)	0.0671 (4)	
N1A	0.7924 (2)	0.91236 (11)	0.01797 (4)	0.0445 (3)	
C1A	0.8636 (2)	0.83620 (14)	−0.07601 (5)	0.0485 (3)	
C2A	0.8931 (3)	0.80560 (16)	−0.12313 (6)	0.0598 (5)	
H2AA	0.9601	0.7419	−0.1301	0.072*	
C3A	0.8235 (3)	0.86924 (18)	−0.15938 (6)	0.0656 (5)	
H3AA	0.8439	0.8479	−0.1907	0.079*	
C4A	0.7240 (3)	0.96424 (17)	−0.15021 (6)	0.0670 (5)	
H4AA	0.6766	1.0063	−0.1750	0.080*	
C5A	0.6955 (3)	0.99621 (16)	−0.10335 (6)	0.0573 (4)	
H5AA	0.6293	1.0604	−0.0969	0.069*	
C6A	0.7647 (2)	0.93353 (13)	−0.06593 (5)	0.0446 (3)	
C7A	0.7319 (2)	0.96829 (14)	−0.01727 (5)	0.0465 (3)	
H7AA	0.6653	1.0328	−0.0116	0.056*	
C8A	0.7652 (2)	0.94552 (13)	0.06567 (5)	0.0416 (3)	
C9A	0.7052 (3)	1.05078 (14)	0.07882 (5)	0.0505 (4)	
H9AA	0.6770	1.1029	0.0556	0.061*	
C10A	0.6870 (3)	1.07866 (16)	0.12636 (6)	0.0555 (4)	
H10A	0.6461	1.1493	0.1345	0.067*	
C11A	0.7287 (3)	1.00352 (17)	0.16175 (5)	0.0521 (4)	
C12A	0.7875 (3)	0.89892 (15)	0.14813 (5)	0.0527 (4)	
H12A	0.8136	0.8470	0.1716	0.063*	
C13A	0.8095 (2)	0.86800 (14)	0.10087 (5)	0.0472 (3)	
C14A	0.7152 (3)	1.0353 (2)	0.21353 (6)	0.0696 (6)	
H14A	0.6420	0.9815	0.2300	0.104*	
H14B	0.6597	1.1073	0.2163	0.104*	
H14C	0.8354	1.0373	0.2272	0.104*	
C15A	0.8789 (4)	0.75441 (16)	0.08827 (7)	0.0703 (6)	
H15A	0.8982	0.7122	0.1168	0.105*	
H15B	0.9922	0.7611	0.0713	0.105*	
H15C	0.7908	0.7172	0.0686	0.105*	
O1B	0.0454 (2)	0.23511 (11)	0.08434 (5)	0.0626 (3)	
N1B	0.1838 (2)	0.08482 (12)	0.14035 (5)	0.0500 (3)	
C1B	0.0999 (2)	0.16993 (14)	0.04804 (6)	0.0487 (4)	
C2B	0.0575 (3)	0.20224 (15)	0.00174 (6)	0.0568 (4)	
H2BA	−0.0090	0.2669	−0.0036	0.068*	
C3B	0.1143 (3)	0.13803 (17)	−0.03601 (6)	0.0628 (5)	
H3BA	0.0864	0.1603	−0.0668	0.075*	
C4B	0.2116 (3)	0.04152 (17)	−0.02891 (6)	0.0620 (5)	
H4BA	0.2514	−0.0001	−0.0548	0.074*	
C5B	0.2499 (3)	0.00667 (16)	0.01675 (6)	0.0551 (4)	
H5BA	0.3128	−0.0596	0.0215	0.066*	
C6B	0.1953 (2)	0.06990 (13)	0.05584 (5)	0.0460 (3)	
C7B	0.2336 (2)	0.03067 (14)	0.10346 (6)	0.0497 (4)	
H7BA	0.2965	−0.0359	0.1073	0.060*	
C8B	0.2118 (2)	0.04277 (15)	0.18674 (5)	0.0494 (4)	
C9B	0.2152 (3)	−0.06991 (16)	0.19735 (6)	0.0582 (4)	
H9BA	0.2036	−0.1216	0.1730	0.070*	
C10B	0.2357 (3)	−0.10637 (17)	0.24352 (6)	0.0660 (5)	
H10B	0.2388	−0.1823	0.2497	0.079*	
C11B	0.2515 (3)	−0.03198 (19)	0.28073 (6)	0.0628 (5)	
C12B	0.2454 (3)	0.07986 (19)	0.26989 (6)	0.0649 (5)	
H12B	0.2544	0.1309	0.2946	0.078*	
C13B	0.2264 (3)	0.11976 (16)	0.22389 (6)	0.0583 (4)	
C14B	0.2744 (4)	−0.0720 (2)	0.33137 (6)	0.0847 (7)	
H14D	0.3425	−0.1402	0.3315	0.127*	
H14E	0.1563	−0.0843	0.3453	0.127*	
H14F	0.3389	−0.0168	0.3494	0.127*	
C15B	0.2263 (5)	0.24280 (17)	0.21427 (8)	0.0910 (9)	
H15D	0.2594	0.2820	0.2427	0.137*	
H15E	0.1065	0.2655	0.2043	0.137*	
H15F	0.3130	0.2593	0.1897	0.137*	
H1OA	0.906 (4)	0.802 (2)	−0.0136 (8)	0.092 (8)*	
H1OB	0.068 (4)	0.201 (2)	0.1120 (8)	0.090 (8)*	

### Atomic displacement parameters (Å^2^)

**Table:**

	*U*^11^	*U*^22^	*U*^33^	*U*^12^	*U*^13^	*U*^23^
O1A	0.0892 (11)	0.0605 (7)	0.0517 (7)	0.0224 (8)	0.0009 (7)	−0.0004 (6)
N1A	0.0474 (7)	0.0463 (6)	0.0398 (6)	−0.0021 (6)	0.0024 (5)	−0.0014 (5)
C1A	0.0499 (8)	0.0489 (8)	0.0468 (8)	0.0004 (7)	0.0029 (7)	−0.0026 (6)
C2A	0.0665 (11)	0.0597 (10)	0.0532 (9)	0.0045 (9)	0.0070 (9)	−0.0120 (8)
C3A	0.0806 (14)	0.0739 (12)	0.0423 (8)	−0.0055 (11)	0.0055 (9)	−0.0090 (8)
C4A	0.0892 (15)	0.0693 (11)	0.0425 (8)	0.0027 (12)	−0.0034 (10)	0.0049 (8)
C5A	0.0730 (12)	0.0515 (8)	0.0475 (8)	0.0059 (9)	−0.0037 (9)	0.0005 (7)
C6A	0.0463 (8)	0.0476 (7)	0.0400 (7)	−0.0031 (7)	0.0008 (6)	−0.0021 (6)
C7A	0.0478 (8)	0.0484 (8)	0.0431 (7)	0.0021 (7)	0.0013 (6)	−0.0035 (6)
C8A	0.0401 (7)	0.0463 (7)	0.0384 (6)	−0.0028 (6)	0.0010 (6)	0.0001 (5)
C9A	0.0607 (9)	0.0476 (8)	0.0433 (7)	0.0029 (8)	−0.0003 (7)	0.0000 (6)
C10A	0.0637 (11)	0.0546 (9)	0.0481 (8)	0.0020 (9)	0.0027 (8)	−0.0074 (7)
C11A	0.0475 (8)	0.0673 (10)	0.0415 (7)	−0.0101 (8)	0.0026 (7)	−0.0053 (7)
C12A	0.0562 (9)	0.0599 (9)	0.0421 (7)	−0.0050 (8)	−0.0023 (7)	0.0070 (7)
C13A	0.0473 (8)	0.0489 (8)	0.0454 (7)	−0.0010 (7)	−0.0018 (7)	0.0022 (6)
C14A	0.0711 (12)	0.0956 (15)	0.0420 (8)	−0.0062 (13)	0.0044 (9)	−0.0117 (9)
C15A	0.0933 (16)	0.0566 (10)	0.0609 (10)	0.0196 (11)	−0.0087 (11)	0.0030 (8)
O1B	0.0783 (9)	0.0510 (6)	0.0586 (7)	0.0045 (7)	0.0039 (7)	−0.0023 (6)
N1B	0.0519 (8)	0.0539 (7)	0.0441 (6)	−0.0028 (7)	0.0005 (6)	0.0005 (6)
C1B	0.0487 (8)	0.0459 (7)	0.0516 (8)	−0.0080 (7)	0.0021 (7)	0.0006 (6)
C2B	0.0579 (10)	0.0540 (9)	0.0586 (9)	−0.0074 (8)	−0.0019 (8)	0.0120 (8)
C3B	0.0713 (12)	0.0709 (11)	0.0462 (8)	−0.0168 (10)	−0.0020 (8)	0.0085 (8)
C4B	0.0735 (12)	0.0650 (10)	0.0474 (8)	−0.0088 (10)	0.0080 (9)	−0.0035 (8)
C5B	0.0594 (10)	0.0540 (8)	0.0519 (8)	−0.0034 (8)	0.0074 (8)	−0.0017 (7)
C6B	0.0465 (8)	0.0477 (8)	0.0437 (7)	−0.0073 (7)	0.0023 (6)	0.0014 (6)
C7B	0.0511 (8)	0.0508 (8)	0.0471 (8)	−0.0019 (7)	0.0017 (7)	0.0023 (7)
C8B	0.0472 (8)	0.0584 (9)	0.0426 (7)	−0.0024 (8)	0.0015 (7)	−0.0010 (7)
C9B	0.0698 (12)	0.0562 (9)	0.0487 (8)	−0.0041 (9)	0.0006 (8)	−0.0015 (7)
C10B	0.0784 (14)	0.0643 (11)	0.0555 (9)	−0.0025 (11)	0.0019 (10)	0.0104 (8)
C11B	0.0599 (11)	0.0837 (13)	0.0449 (8)	0.0007 (11)	0.0037 (8)	0.0048 (8)
C12B	0.0729 (13)	0.0749 (11)	0.0470 (8)	0.0052 (11)	0.0024 (9)	−0.0097 (8)
C13B	0.0664 (11)	0.0595 (9)	0.0490 (8)	0.0038 (9)	0.0036 (8)	−0.0060 (7)
C14B	0.0918 (17)	0.1126 (19)	0.0496 (10)	0.0030 (17)	0.0001 (11)	0.0142 (11)
C15B	0.143 (3)	0.0597 (11)	0.0709 (12)	0.0069 (16)	0.0003 (17)	−0.0125 (10)

### Geometric parameters (Å, °)

**Table:**

O1A—C1A	1.346 (2)	O1B—C1B	1.349 (2)
O1A—H1OA	0.88 (3)	O1B—H1OB	0.89 (2)
N1A—C7A	1.278 (2)	N1B—C7B	1.279 (2)
N1A—C8A	1.4155 (18)	N1B—C8B	1.416 (2)
C1A—C2A	1.394 (2)	C1B—C2B	1.395 (2)
C1A—C6A	1.405 (2)	C1B—C6B	1.408 (2)
C2A—C3A	1.374 (3)	C2B—C3B	1.378 (3)
C2A—H2AA	0.9300	C2B—H2BA	0.9300
C3A—C4A	1.379 (3)	C3B—C4B	1.376 (3)
C3A—H3AA	0.9300	C3B—H3BA	0.9300
C4A—C5A	1.390 (2)	C4B—C5B	1.382 (2)
C4A—H4AA	0.9300	C4B—H4BA	0.9300
C5A—C6A	1.391 (2)	C5B—C6B	1.396 (2)
C5A—H5AA	0.9300	C5B—H5BA	0.9300
C6A—C7A	1.453 (2)	C6B—C7B	1.449 (2)
C7A—H7AA	0.9300	C7B—H7BA	0.9300
C8A—C9A	1.390 (2)	C8B—C9B	1.388 (3)
C8A—C13A	1.399 (2)	C8B—C13B	1.401 (2)
C9A—C10A	1.387 (2)	C9B—C10B	1.380 (2)
C9A—H9AA	0.9300	C9B—H9BA	0.9300
C10A—C11A	1.379 (3)	C10B—C11B	1.383 (3)
C10A—H10A	0.9300	C10B—H10B	0.9300
C11A—C12A	1.384 (3)	C11B—C12B	1.380 (3)
C11A—C14A	1.511 (2)	C11B—C14B	1.514 (2)
C12A—C13A	1.391 (2)	C12B—C13B	1.389 (2)
C12A—H12A	0.9300	C12B—H12B	0.9300
C13A—C15A	1.500 (2)	C13B—C15B	1.505 (3)
C14A—H14A	0.9600	C14B—H14D	0.9600
C14A—H14B	0.9600	C14B—H14E	0.9600
C14A—H14C	0.9600	C14B—H14F	0.9600
C15A—H15A	0.9600	C15B—H15D	0.9600
C15A—H15B	0.9600	C15B—H15E	0.9600
C15A—H15C	0.9600	C15B—H15F	0.9600
			
C1A—O1A—H1OA	109.1 (17)	C1B—O1B—H1OB	110.0 (17)
C7A—N1A—C8A	122.67 (14)	C7B—N1B—C8B	121.75 (15)
O1A—C1A—C2A	118.90 (16)	O1B—C1B—C2B	118.72 (16)
O1A—C1A—C6A	121.63 (14)	O1B—C1B—C6B	121.66 (15)
C2A—C1A—C6A	119.47 (15)	C2B—C1B—C6B	119.61 (16)
C3A—C2A—C1A	120.19 (17)	C3B—C2B—C1B	119.84 (18)
C3A—C2A—H2AA	119.9	C3B—C2B—H2BA	120.1
C1A—C2A—H2AA	119.9	C1B—C2B—H2BA	120.1
C2A—C3A—C4A	121.21 (16)	C4B—C3B—C2B	121.10 (17)
C2A—C3A—H3AA	119.4	C4B—C3B—H3BA	119.4
C4A—C3A—H3AA	119.4	C2B—C3B—H3BA	119.4
C3A—C4A—C5A	119.07 (18)	C3B—C4B—C5B	119.74 (18)
C3A—C4A—H4AA	120.5	C3B—C4B—H4BA	120.1
C5A—C4A—H4AA	120.5	C5B—C4B—H4BA	120.1
C4A—C5A—C6A	120.98 (18)	C4B—C5B—C6B	120.70 (18)
C4A—C5A—H5AA	119.5	C4B—C5B—H5BA	119.6
C6A—C5A—H5AA	119.5	C6B—C5B—H5BA	119.6
C5A—C6A—C1A	119.08 (14)	C5B—C6B—C1B	118.96 (15)
C5A—C6A—C7A	119.90 (15)	C5B—C6B—C7B	119.78 (16)
C1A—C6A—C7A	121.02 (14)	C1B—C6B—C7B	121.24 (15)
N1A—C7A—C6A	121.58 (15)	N1B—C7B—C6B	122.04 (16)
N1A—C7A—H7AA	119.2	N1B—C7B—H7BA	119.0
C6A—C7A—H7AA	119.2	C6B—C7B—H7BA	119.0
C9A—C8A—C13A	119.42 (14)	C9B—C8B—C13B	118.89 (16)
C9A—C8A—N1A	123.64 (14)	C9B—C8B—N1B	123.37 (15)
C13A—C8A—N1A	116.88 (14)	C13B—C8B—N1B	117.64 (16)
C10A—C9A—C8A	120.51 (15)	C10B—C9B—C8B	121.00 (17)
C10A—C9A—H9AA	119.7	C10B—C9B—H9BA	119.5
C8A—C9A—H9AA	119.7	C8B—C9B—H9BA	119.5
C11A—C10A—C9A	121.19 (17)	C9B—C10B—C11B	121.15 (18)
C11A—C10A—H10A	119.4	C9B—C10B—H10B	119.4
C9A—C10A—H10A	119.4	C11B—C10B—H10B	119.4
C10A—C11A—C12A	117.65 (15)	C12B—C11B—C10B	117.41 (17)
C10A—C11A—C14A	121.14 (18)	C12B—C11B—C14B	121.42 (19)
C12A—C11A—C14A	121.19 (17)	C10B—C11B—C14B	121.2 (2)
C11A—C12A—C13A	122.96 (16)	C11B—C12B—C13B	123.12 (18)
C11A—C12A—H12A	118.5	C11B—C12B—H12B	118.4
C13A—C12A—H12A	118.5	C13B—C12B—H12B	118.4
C12A—C13A—C8A	118.23 (15)	C12B—C13B—C8B	118.42 (18)
C12A—C13A—C15A	120.59 (15)	C12B—C13B—C15B	120.53 (18)
C8A—C13A—C15A	121.18 (14)	C8B—C13B—C15B	121.03 (16)
C11A—C14A—H14A	109.5	C11B—C14B—H14D	109.5
C11A—C14A—H14B	109.5	C11B—C14B—H14E	109.5
H14A—C14A—H14B	109.5	H14D—C14B—H14E	109.5
C11A—C14A—H14C	109.5	C11B—C14B—H14F	109.5
H14A—C14A—H14C	109.5	H14D—C14B—H14F	109.5
H14B—C14A—H14C	109.5	H14E—C14B—H14F	109.5
C13A—C15A—H15A	109.5	C13B—C15B—H15D	109.5
C13A—C15A—H15B	109.5	C13B—C15B—H15E	109.5
H15A—C15A—H15B	109.5	H15D—C15B—H15E	109.5
C13A—C15A—H15C	109.5	C13B—C15B—H15F	109.5
H15A—C15A—H15C	109.5	H15D—C15B—H15F	109.5
H15B—C15A—H15C	109.5	H15E—C15B—H15F	109.5
			
O1A—C1A—C2A—C3A	178.71 (19)	O1B—C1B—C2B—C3B	178.93 (17)
C6A—C1A—C2A—C3A	−1.0 (3)	C6B—C1B—C2B—C3B	−2.1 (3)
C1A—C2A—C3A—C4A	0.1 (3)	C1B—C2B—C3B—C4B	0.5 (3)
C2A—C3A—C4A—C5A	0.6 (3)	C2B—C3B—C4B—C5B	1.5 (3)
C3A—C4A—C5A—C6A	−0.4 (3)	C3B—C4B—C5B—C6B	−1.8 (3)
C4A—C5A—C6A—C1A	−0.4 (3)	C4B—C5B—C6B—C1B	0.1 (3)
C4A—C5A—C6A—C7A	−179.54 (19)	C4B—C5B—C6B—C7B	178.76 (17)
O1A—C1A—C6A—C5A	−178.57 (17)	O1B—C1B—C6B—C5B	−179.27 (16)
C2A—C1A—C6A—C5A	1.1 (3)	C2B—C1B—C6B—C5B	1.8 (2)
O1A—C1A—C6A—C7A	0.5 (3)	O1B—C1B—C6B—C7B	2.1 (2)
C2A—C1A—C6A—C7A	−179.75 (17)	C2B—C1B—C6B—C7B	−176.81 (16)
C8A—N1A—C7A—C6A	178.82 (15)	C8B—N1B—C7B—C6B	176.07 (15)
C5A—C6A—C7A—N1A	179.56 (17)	C5B—C6B—C7B—N1B	−178.89 (17)
C1A—C6A—C7A—N1A	0.5 (3)	C1B—C6B—C7B—N1B	−0.3 (3)
C7A—N1A—C8A—C9A	−13.7 (2)	C7B—N1B—C8B—C9B	−29.7 (3)
C7A—N1A—C8A—C13A	169.08 (15)	C7B—N1B—C8B—C13B	153.98 (18)
C13A—C8A—C9A—C10A	−0.8 (3)	C13B—C8B—C9B—C10B	−1.1 (3)
N1A—C8A—C9A—C10A	−177.94 (16)	N1B—C8B—C9B—C10B	−177.34 (19)
C8A—C9A—C10A—C11A	0.4 (3)	C8B—C9B—C10B—C11B	0.7 (4)
C9A—C10A—C11A—C12A	−0.6 (3)	C9B—C10B—C11B—C12B	0.2 (4)
C9A—C10A—C11A—C14A	177.81 (18)	C9B—C10B—C11B—C14B	−179.9 (2)
C10A—C11A—C12A—C13A	1.3 (3)	C10B—C11B—C12B—C13B	−0.7 (4)
C14A—C11A—C12A—C13A	−177.09 (19)	C14B—C11B—C12B—C13B	179.3 (2)
C11A—C12A—C13A—C8A	−1.7 (3)	C11B—C12B—C13B—C8B	0.3 (3)
C11A—C12A—C13A—C15A	178.23 (19)	C11B—C12B—C13B—C15B	−178.2 (3)
C9A—C8A—C13A—C12A	1.4 (2)	C9B—C8B—C13B—C12B	0.6 (3)
N1A—C8A—C13A—C12A	178.78 (15)	N1B—C8B—C13B—C12B	177.06 (18)
C9A—C8A—C13A—C15A	−178.55 (18)	C9B—C8B—C13B—C15B	179.1 (2)
N1A—C8A—C13A—C15A	−1.2 (2)	N1B—C8B—C13B—C15B	−4.4 (3)

### Hydrogen-bond geometry (Å, °)

**Table:**

*D*—H···*A*	*D*—H	H···*A*	*D*···*A*	*D*—H···*A*
O1A—H1OA···N1A	0.88 (2)	1.80 (2)	2.5854 (19)	147 (2)
O1B—H1OB···N1B	0.90 (2)	1.82 (2)	2.604 (2)	145 (2)
C5A—H5AA···O1B^i^	0.93	2.56	3.455 (2)	162

Symmetry codes: (i) *x*+1/2, −*y*+3/2, −*z*.
